# Uncovering differential gene expression between mtRNA-positive and -negative osteosarcoma cells: implications beyond mitochondrial function

**DOI:** 10.3389/fgene.2025.1588581

**Published:** 2025-09-17

**Authors:** Xiaoyuan Meng, Zhongcheng Han, Wuerkan Yeerken, Zhigang Wang, Le Ma, Hongbo Liu

**Affiliations:** ^1^ Department of Orthopedics, People’s Hospital of Xinjiang Uygur Autonomous Region, Urumqi, China; ^2^ Department of Oncology, People’s Hospital of Xinjiang Uygur Autonomous Region, Urumqi, China; ^3^ Operating Theater, People’s Hospital of Xinjiang Uygur Autonomous Region, Urumqi, China

**Keywords:** osteosarcoma, mtRNA, hub genes, bioinformatics analysis, COL1A1

## Abstract

**Background:**

Long-term clinical outcomes for patients with osteosarcoma have shown little improvement over the past few decades. Identifying novel molecular targets to inhibit osteosarcoma cell growth remains an urgent challenge.

**Methods:**

Explore the function of mtRNA in the occurrence and development of osteosarcoma utilizing bioinformatics analysis of the Gene Expression Omnibus (GEO) microarray dataset. The Network Analyst tool was used to analyze GSE73120. Differentially expressed genes (DEGs) were identified using GEO2R and analyzed using NetworkAnalyst. Gene Ontology (GO) and Kyoto Encyclopedia of Genes and Genomes (KEGG) enrichment analysis were performed using Metascape and WebGestalt. A protein–protein interaction (PPI) network was constructed through the STRING database and visualized with Cytoscape. The MCODE algorithm was used to identify key modules, and CytoHubba was applied to determine hub genes. Validation of hub genes was conducted using the GEPIA database.

**Results:**

A total of 104 DEGs were identified, including 89 upregulated and 15 downregulated genes. GO and KEGG analyses revealed that these DEGs were enriched in pathways related to connective tissue development, collagen trimer, and extracellular matrix structural components. The PPI network analysis identified seven hub genes. Among them, COL1A1, PDGFRB, and SPARC were confirmed as sarcoma-related genes using the GEPIA database.

**Conclusion:**

Our findings suggest that COL1A1, PDGFRB, and SPARC may be involved in mtRNA-driven tumorigenesis and could serve as promising therapeutic targets for osteosarcoma.

## 1 Introduction

Osteosarcoma arises from primitive mesenchymal cells that produce osteoid or immature bone. It is the most common primary malignant bone tumor in adolescents and young adults. Incidence peaks between ages 0 and 24, reaching approximately 4.4 cases per million annually in this age group (Mirabello et al., 2009; Ge et al., 2022; Guillon et al., 2011; Xing et al., 2014). Although advanced progress has been made in many fields, such as pathophysiology, tumor immunology, and genetics, long-term clinical outcomes for patients with osteosarcoma have shown no significant improvement during the past few decades (Yu and Yao, 2024; Misaghi et al., 2018; Bernthal et al., 2012). Hence, finding a novel target to inhibit osteosarcoma cell growth is an urgent problem to be solved.

In human cells, mitochondrial RNA (mtRNA) codes 13 essential proteins of the oxidative phosphorylation (OXPHOS) system, along with 22 mitochondrial tRNAs (mt-tRNAs) and 2 mitochondrial RNAs (mt-RNAs) (Taanman, 1999; Jedynak-Slyvka et al., 2021). Crucially, disruption in mtRNA processing or degradation, such as in RNase-mediated cleavage of polycistronic transcripts or failure to remove aberrant RNAs, can impair mitochondrial function at several levels (Wallace, 2012). Poorly processed or unstable mt-mRNAs and mt-tRNAs impair the translation of the 13 mitochondrial-encoded OXPHOS subunits ND1-6, COXI-III, ATP6/8, and CYB, reducing their levels and preventing proper assembly of complexes I–V, particularly I, IV, and V (11). This disruption collapses the proton gradient, sharply diminishing ATP synthesis, and forcing cells to upregulate glycolysis and alternative substrate-level phosphorylation (Seyfried et al., 2020). In cancers such as osteosarcoma, this metabolic shift supports proliferation under hypoxia while increasing reactive oxygen species (ROS), DNA damage, and survival signaling (Tufail et al., 2024). Although similar mtRNA-driven oncogenic mechanisms have been documented in lung and breast cancers (Shapovalov et al., 2011), their specific effects on OXPHOS integrity, ATP production, and tumor cell survival in osteosarcoma remain to be elucidated.

To investigate the role of mtRNA in osteosarcoma, we analyzed the GSE73120 dataset from the Gene Expression Omnibus (GEO) database. This dataset includes gene expression profiles from 143B osteosarcoma cells containing mitochondrial DNA (mtDNA) and their rho-zero counterparts lacking mtDNA. Different expression analysis was performed using the GEO2R tool to identify genes with significant expression differences between the two groups. Gene Ontology (GO) and Kyoto Encyclopedia of Genes and Genomes (KEGG) pathway enrichment analyses were conducted to elucidate the biological functions and pathways associated with the differentially expressed genes (DEGs). A protein–protein interaction (PPI) network was constructed, and the hub genes were acquired using the Maximal Clique Centrality (MCC) and Molecular Complex Detection (MOCDE) algorithm. Finally, the gene expression profiling interactive analysis (GEPIA) database was utilized to assess the expression levels and prognostic significance of the hub genes in sarcoma.

## 2 Materials and methods

### 2.1 Data sources

We queried GEO with “osteosarcoma” and “*Homo sapiens*” and retrieved dataset GSE73120 (Lee et al., 2016), which contains gene expression profiles from 143B osteosarcoma tumors either harboring their endogenous mtDNA or their rho-zero counterparts lacking mtDNA. The data were generated using the GPL10558 Illumina HumanHT-12 V4.0 expression beadchip, enabling genome-wide transcriptomic analysis across both tumor types (Lee et al., 2016).

### 2.2 Differential expression analysis

DEGs were identified using GEO2R (https://www.ncbi.nlm.nih.gov/geo/geo2r/), a web-based tool that applies the limma and GEOquery R packages to compare gene expression between the mtDNA-proficient (OM) and mtDNA-deficient (WOM) 143B osteosarcoma groups (Love et al., 2014). We considered genes to be significantly differentially expressed if they met the adjusted p-value <0.05 and |log2 fold change (FC)| >1.

### 2.3 Enrichment analyses

Functional enrichment of DEGs was performed using Metascape (https://metascape.org/gp/index.html#/main/step1) and WebGestalt (http://www.webgestalt.org/), which integrate the GO and KEGG pathway databases (Zhou et al., 2019). The visualization of the results of the enrichment analyses used the Xiantao website (https://www.xiantao.love/). An adjusted p-value <0.05 was considered significant.

### 2.4 PPI network analysis and the hub genes

The STRING (https://cn.string-db.org/) database was used to construct a PPI network, and Cytoscape v.3.7.1 software was used to visualize the network. A combined score >0.4 was the standard to identify the significant PPIs. Hub genes were identified using the CytoHubba plugin’s MCC algorithm, and network modules were extracted using MCODE.

### 2.5 Hub gene expression in sarcoma

Hub genes were validated using the GEPIA database (http://gepia.cancer-pku.cn/index.html), which integrates The Cancer Genome Analysis (TCGA) and genotype tissue expression (GTEx) data. We compared expression levels between sarcoma (SARC) and matched normal tissues, assessed overall survival associations, and examined inter-gene correlations using Spearman analysis.

### 2.6 Statistical analysis

All statistical evaluations were performed using R (version 4.1.0). GEO2R and enrichment tools applied built-in corrections (Benjamini–Hochberg FDR). Spearman’s rank correlation was employed for gene co-expression analysis. A two-tailed p-value <0.05 was considered statistically significant throughout.

## 3 Results

### 3.1 Identification of DEGs in GSE73120

**FIGURE 1:**
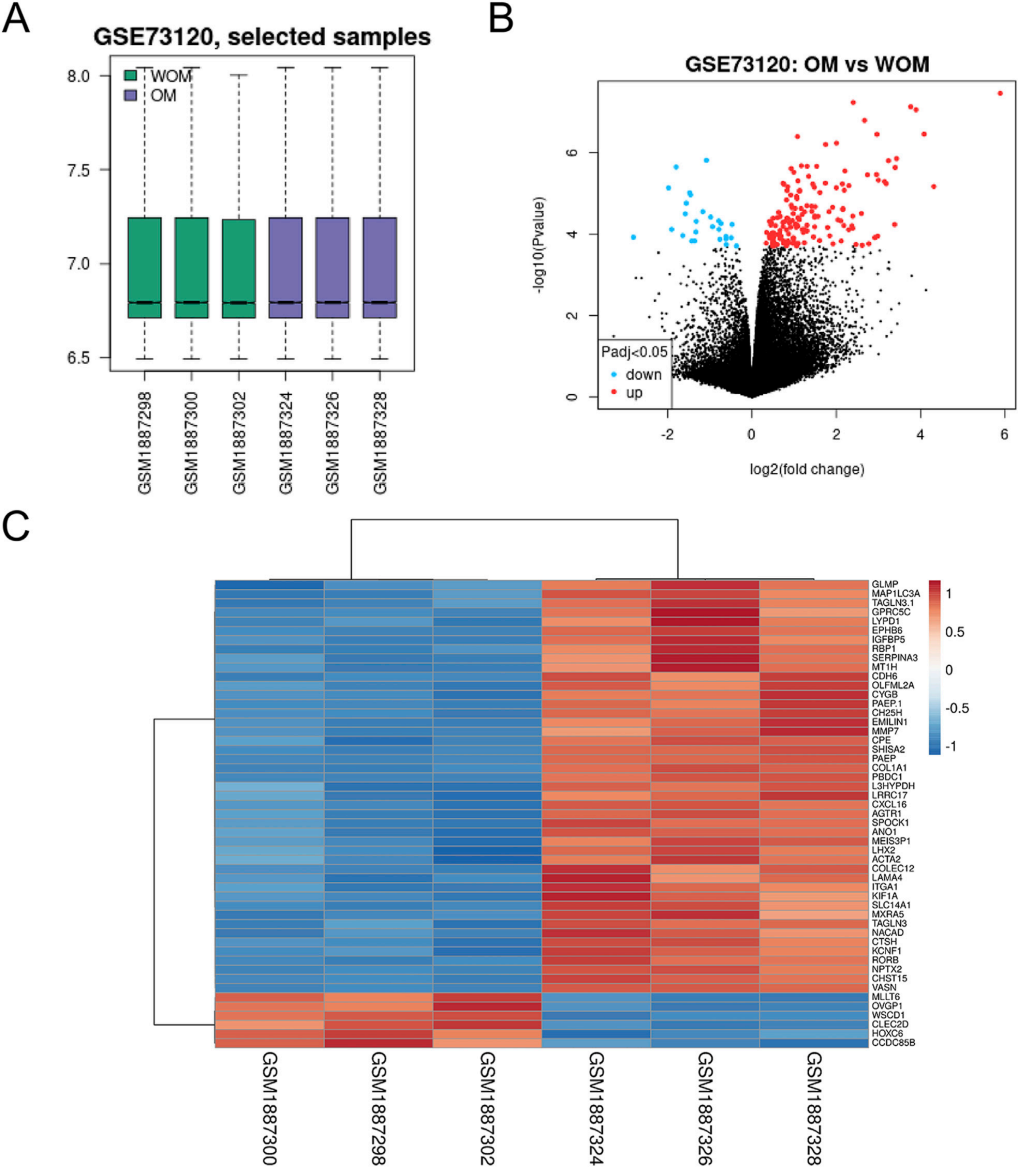
Identification of DEGs in the GSE73120 osteosarcoma dataset. **(A)** The normalization of the osteosarcoma dataset; **(B)** volcano plot of gene expression profile data in WOM and OM samples; **(C)** the heat map of the dataset demonstrated distinguished features between WOM and OM samples.

Analysis of GSE73120 via GEO2R, with standard standardization (Figure 1A), identified 104 DEGs, comprising 89 upregulated and 15 downregulated genes in mtDNA-proficient versus -deficient 143B osteosarcoma cells (Supplementary Table S1). A volcano plot and a heatmap (Figures 1B,C) demonstrated clear separation between mtRNA-competent and mtRNA-deficient samples based on mitochondrial gene expression patterns.

### 3.2 Functional enrichment analysis of mitochondria-related DEGs

**FIGURE 2:**
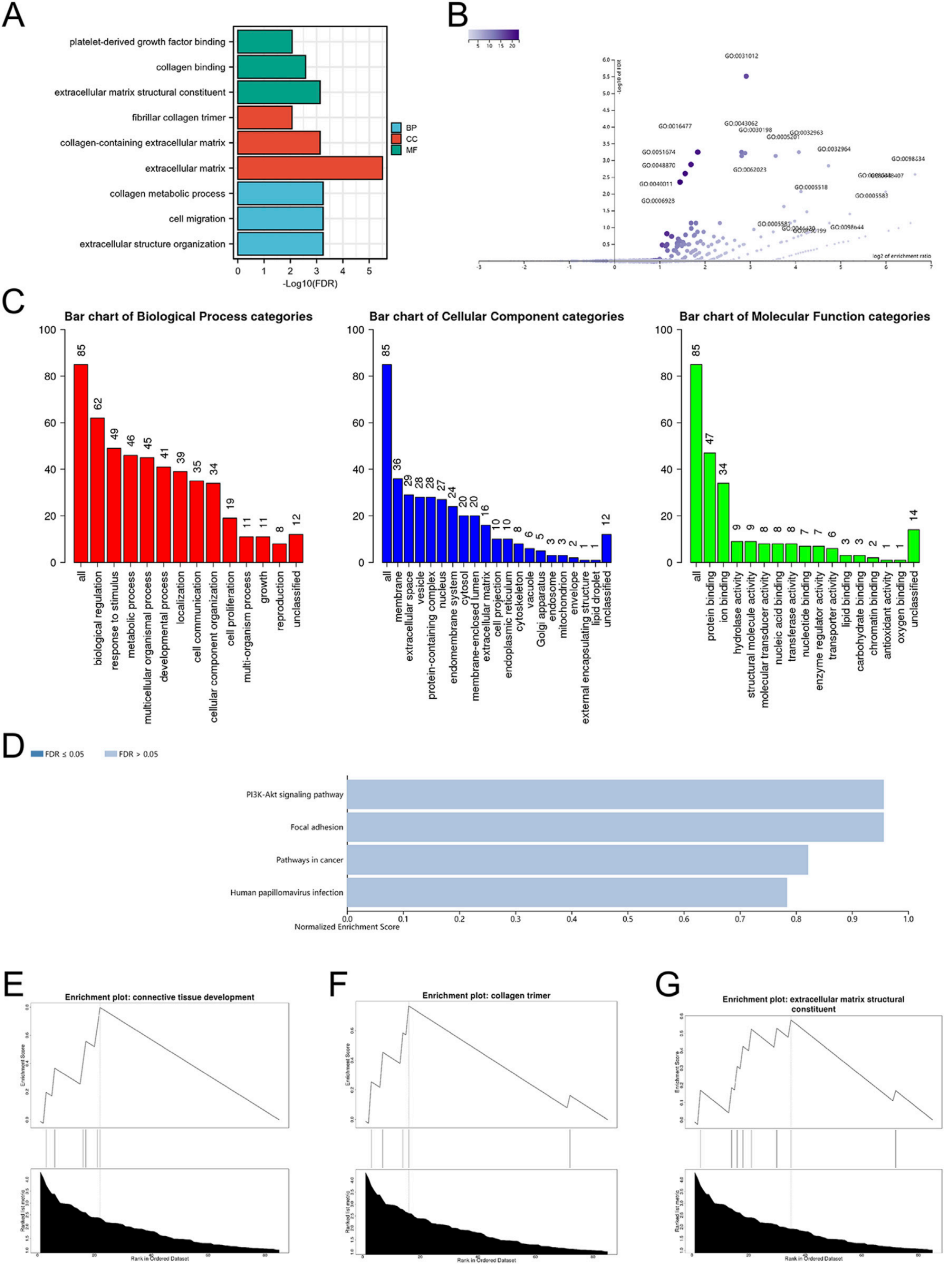
The enrichment pathway analysis of DEGs. **(A)** The significant GO pathways enriched by DEGs; volcano plot **(B)** and bar graph **(C)** of the GO enrichment pathway analysis of DEGs using WebGestalt; **(D)** bar graph of the KEGG enrichment pathway analysis of DEGs using WebGestalt; the gene set enrichment analysis of DEGs using WebGestalt **(E–G)**.

**FIGURE 3:**
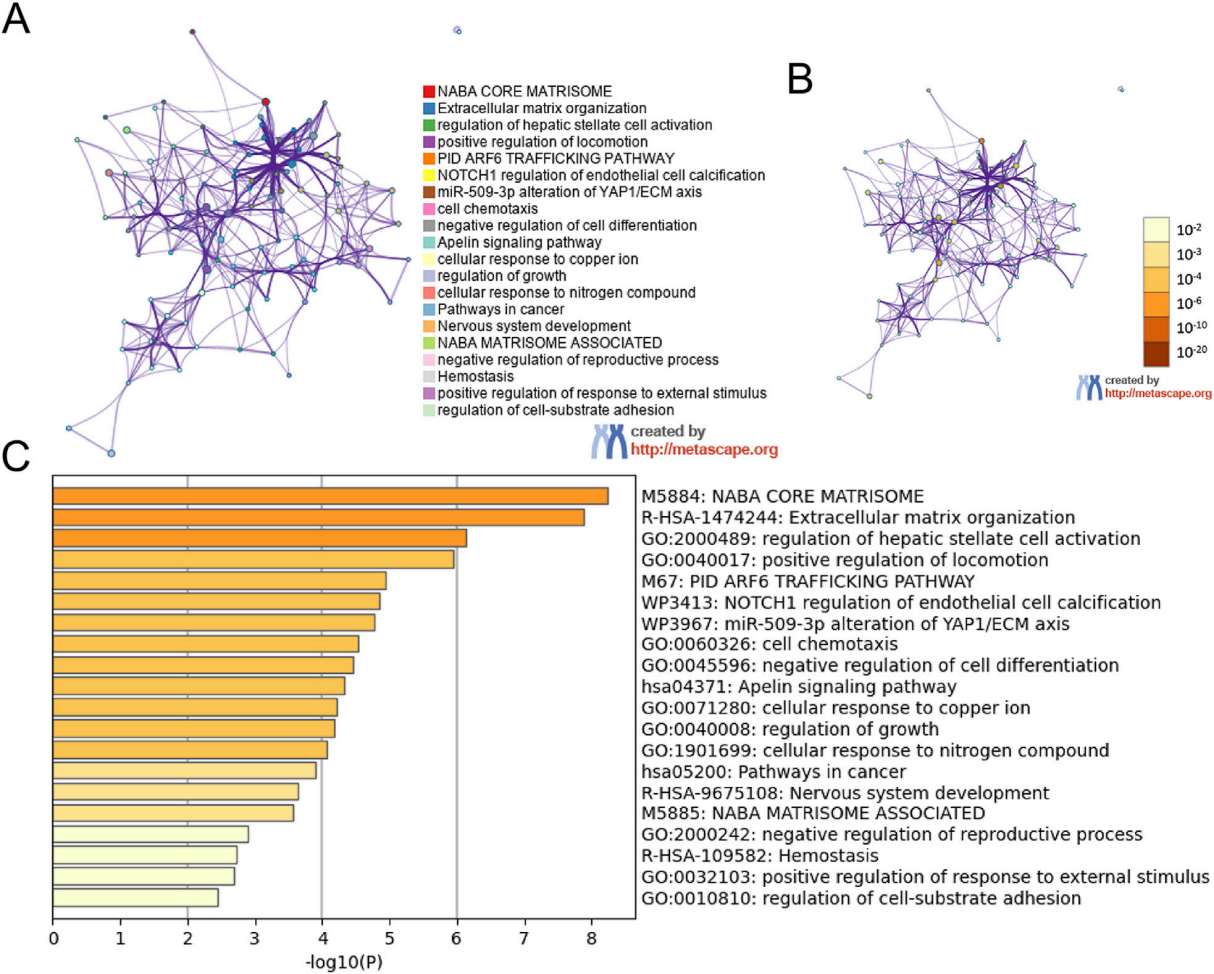
The enrichment pathway analysis of DEGs. The pathways network of DEGs using Metascape: **(A)** colored by cluster ID, where nodes that share the same cluster ID are typically close to each other; **(B)** colored by p-value, where terms containing more genes tend to have a more significant p-value; **(C)** the enrichment pathway analysis of DEGs using Metascape.

By uploading the mitochondria-related DEGs into the Xiantao webpage and Metascape, the results of the enrichment analyses indicated that the mitochondria-related DEGs were involved in extracellular matrix structural constituents, regulation of hepatic stellate cell activation, biological regulation, membrane, and protein binding (Figures 2A–D, 3A–C; Supplementary Table S2). Notably, gene set enrichment analysis consistently identified terms related to extracellular matrix and collagen formation (Figures 2E–G, 3A–C).

### 3.3 PPI analysis of mitochondria-related DEGs

**FIGURE 4:**
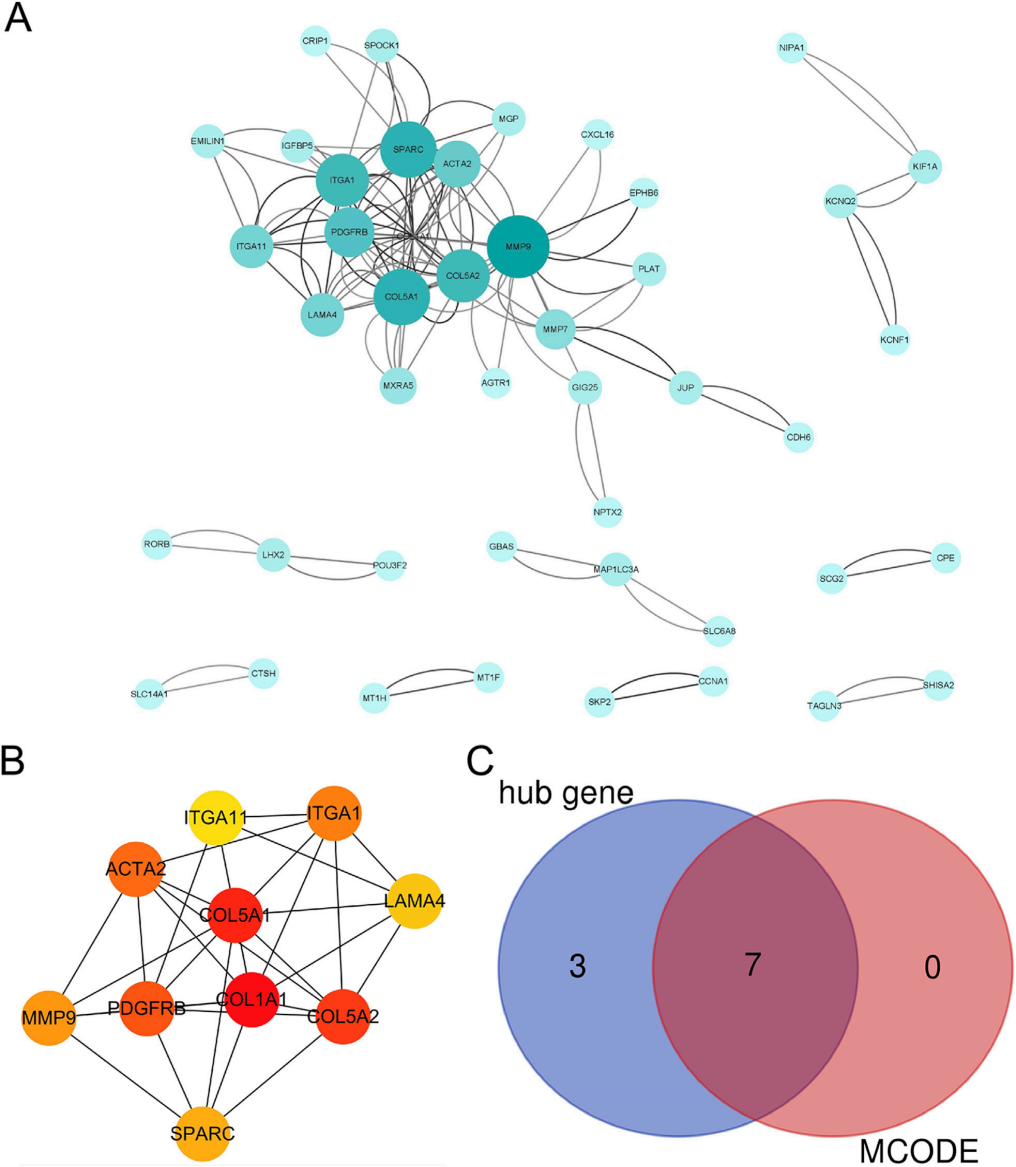
The PPI analysis of DEGs. **(A)** The PPI analysis of ferroptosis-related DEGs, colored by degree centrality; **(B)** the top 10 DEGs using MCC; **(C)** a Venn diagram of DEGs using the MCC and MCODE analysis module.

The PPI network was constructed using STRING and Cytoscape and revealed a densely interconnected module (Figure 4A; Supplementary Table S3), from which CytoHubba’s MCC algorithm ranked COL5A1, ITGA1, PDGFRB, SPARC, COL1A1, ITGA11, LAMA4, ACTA2, COL5A2, and MMP9 as top hub genes (Figure 4B). Further MCODE clustering confirmed COL5A1, ITGA1, PDGFRB, SPARC, COL1A1, LAMA4, and MMP9 as members of a core mitochondrial extracellular matrix-related module. A Venn diagram illustrated the overlap among these candidate hubs (Figure 4C).

### 3.4 Validation of hub gene expression in sarcoma

**FIGURE 5:**
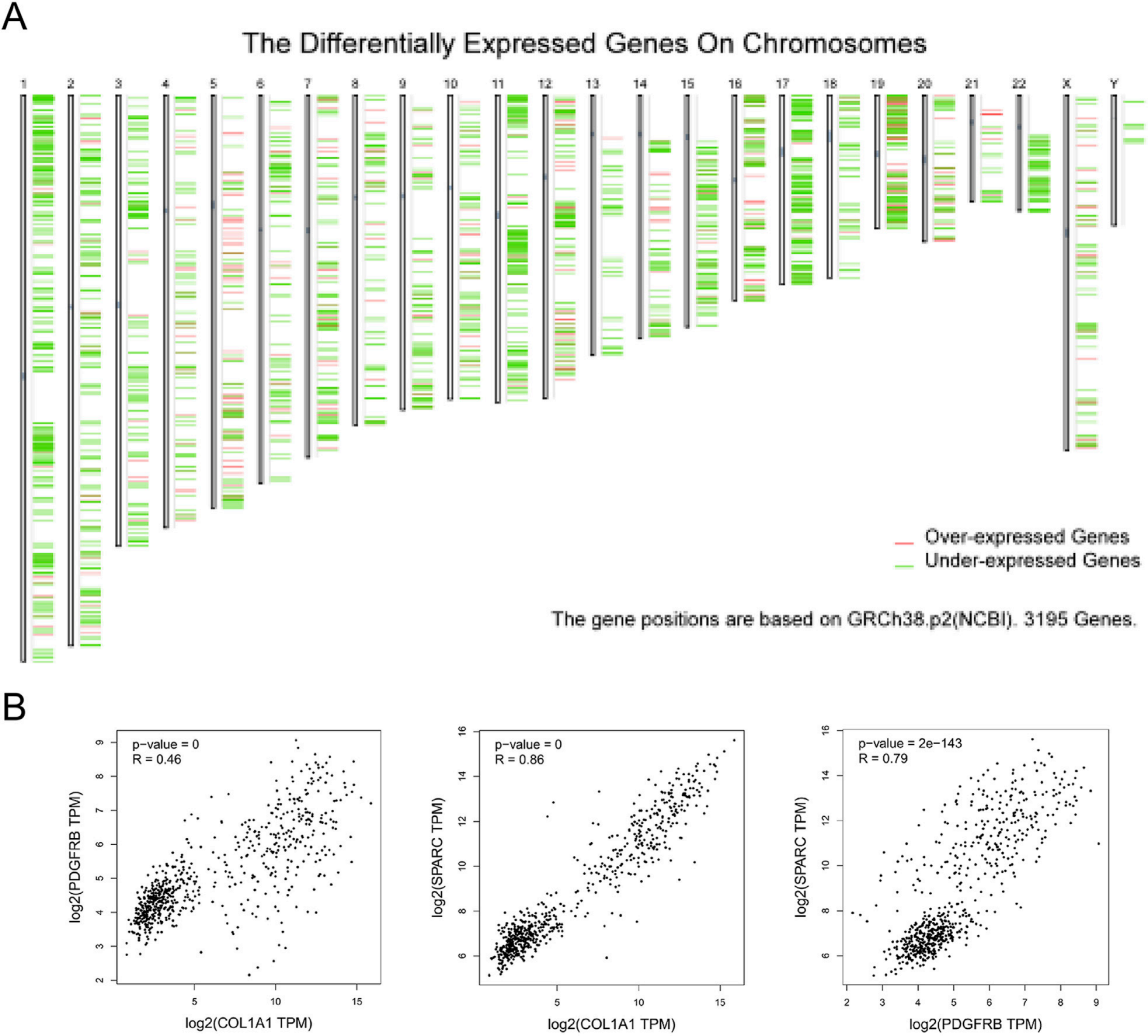
The distribution of differently expressed genes and their correlation in SARC. **(A)** The distribution of differently expressed genes on chromosomes in SARC; **(B)** the correlation among three hub genes.

**FIGURE 6:**
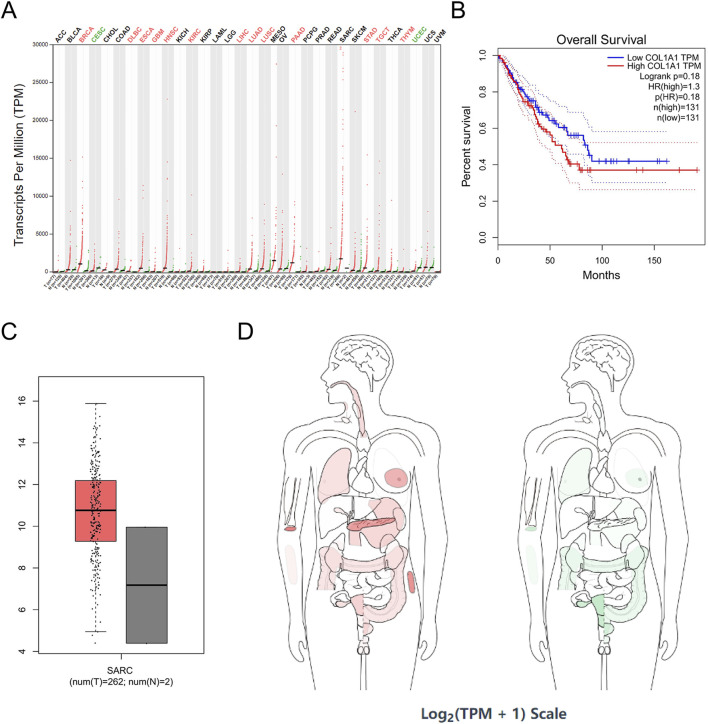
COL1A1 expression in cancer. **(A)** COL1A1 expression profile across all tumor samples and paired normal tissues in TCGA; **(B)** the overall survival analysis of COL1A1 expression in SARC; **(C)** expression of COL1A1 in SARC on box plots; **(D)** the median expression of tumor and normal samples in BodyMap. Red, tumor samples; green, normal samples.

Gene expression data for sarcoma were extracted and analyzed using the GEPIA database (Figure 5A). Through this analysis, COL1A1, PDGFRB, and SPARC were identified as sarcoma-related genes. Specifically, COL1A1 was found to be highly expressed in 13 tumor types, including breast invasive carcinoma (BRCA), diffuse large B-cell lymphoma (DLBC), esophageal carcinoma (ESCA), glioblastoma multiforme (GBM), head and neck squamous cell carcinoma (HNSC), kidney chromophobe (KICH), liver hepatocellular carcinoma (LIHC), lung adenocarcinoma (LUAD), lung squamous cell carcinoma (LUSC), pancreatic adenocarcinoma (PAAD), stomach adenocarcinoma (STAD), thyroid carcinoma (THCA), and thymoma (THYM). In contrast, low expression of COL1A1 was observed in cervical squamous cell carcinoma and endocervical adenocarcinoma (CESC), as well as uterine corpus endometrial carcinoma (UCEC) (Figure 6A).

**FIGURE 7 F7:**
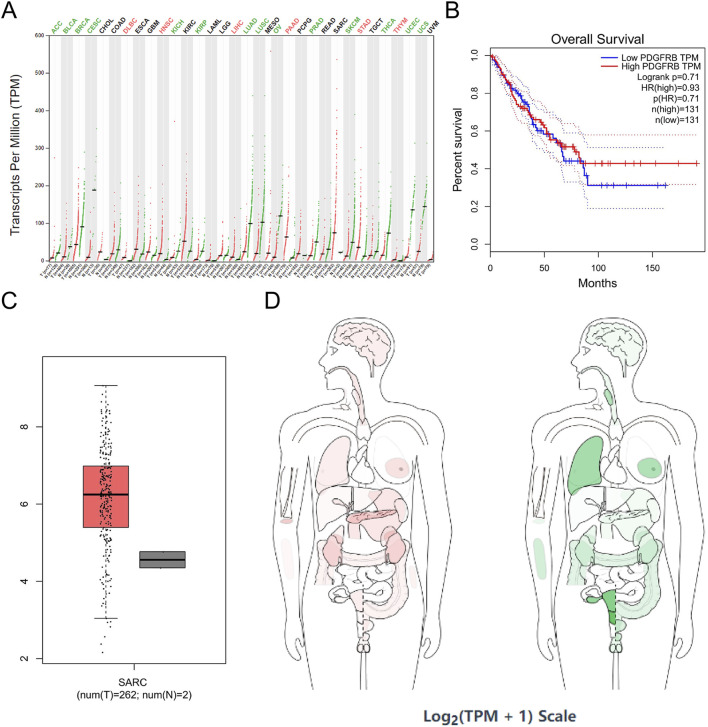
PDGFRB expression in cancer. **(A)** PDGFRB expression profile across all tumor samples and paired normal tissues in TCGA; **(B)** the overall survival analysis of PDGFRB expression in SARC; **(C)** expression of PDGFRB in SARC on box plots; **(D)** the median expression of tumor and normal samples in BodyMap. Red, tumor samples; green, normal samples.

PDGFRB exhibited high expression in six tumor cell lines, including DLBC, HNSC, LIHC, PAAD, STAD, and THYM, and low expression in 12 tumor cell lines, including adrenocortical carcinoma (ACC), bladder urothelial carcinoma (BLCA), KICH, kidney renal papillary cell carcinoma (KIRP), LUAD, LUSC, ovarian serous cystadenocarcinoma (OV), prostate adenocarcinoma (PRAD), skin cutaneous melanoma (SKCM), THCA, UCEC, and uterine carcinosarcoma (UCS) (Figure 7A).

**FIGURE 8 F8:**
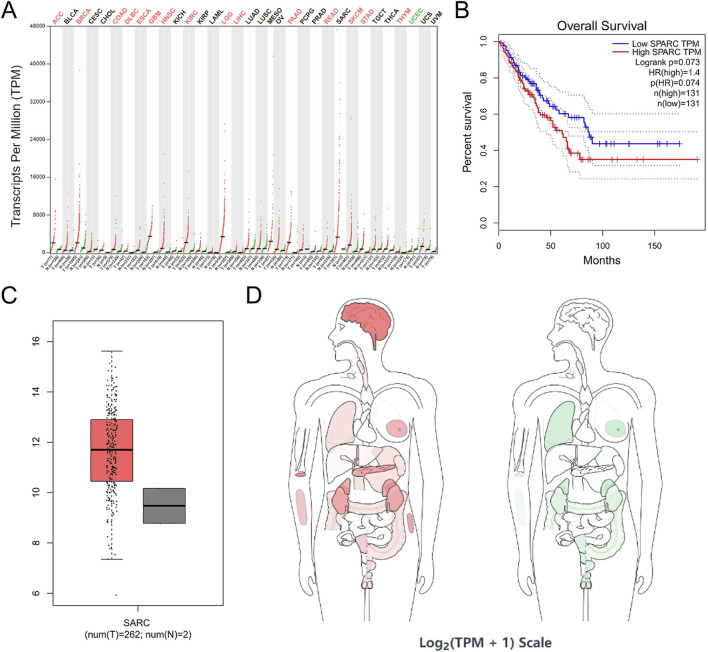
SPARC expression in cancer. (A) SPARC expression profile across all tumor samples and paired normal tissues in TCGA; (B) the overall survival analysis of SPARC expression in SARC; (C) expression of SPARC in SARC on box plots; (D) the median expression of tumor and normal samples in BodyMap. Red, tumor samples; green, normal samples.

SPARC was highly expressed in 15 tumor cell lines, such as ACC, BRCA, colon adenocarcinoma (COAD), DLBC, ESCA, GBM, HNSC, kidney renal clear cell carcinoma (KIRC), brain lower-grade glioma (LGG), LIHC, PAAD, rectum adenocarcinoma (READ), SKCM, STAD, and THYM, and was expressed at a low level in UCEC (Figure 8A). In addition, correlation analysis among COL1A1, PDGFRB, and SPARC revealed a significant interrelationship (Figure 5B). However, survival analysis indicated that the expression levels of these genes did not significantly influence the overall survival (OS) in sarcoma patients (Figures 6B,7B,8B, [Fig F7], [Fig F8]). Moreover, analysis of paired tumor and normal tissue samples showed no significant differential expression of COL1A1, PDGFRB, and SPARC (Figures 6C,7C,8C,D, [Fig F7], [Fig F8]).

## 4 Discussion

Mitochondria, the cell’s powerhouses, generate ATP through oxidative OXPHOS, relying critically on mitochondrial RNA (mtRNA) for expression of OXPHOS components. Notably, osteosarcoma is characterized by increased OXPHOS and ATP production, which correlate with increased cell migration and aggressive phenotypes (Huan et al., 2024; Han et al., 2021; Yu et al., 2021). Han et al. reported that RNA polymerase mitochondrial (POLRMT), essential for mtRNA transcription, is significantly upregulated in osteosarcoma, and its inhibition impairs mtRNA synthesis, mitochondrial function, and tumor growth (Huan et al., 2024; Han et al., 2021; Yu et al., 2021; Li et al., 2023; Kong et al., 2024). To further investigate the role of mtRNA in osteosarcoma, we analyzed the mitochondria-related dataset GSE73120, aiming to clarify how mtRNA abundance influences mitochondrial dynamics and tumor progression.

Analysis of the GSE73120 dataset identified 104 differentially expressed genes, comprising 89 upregulated and 15 downregulated transcripts. GO and KEGG analyses revealed that mitochondria-related DEGs are enriched in biological processes and cellular components such as connective tissue development, collagen trimers, and extracellular matrix (ECM) structural constituents. A subsequent PPI network, analyzed using MCC and MCODE algorithms, highlighted COL5A1, ITGA1, PDGFRB, SPARC, COL1A1, LAMA4, and MMP9 as central hub genes. We further validated these candidates via the GEPIA database, narrowing down to COL1A1, PDGFRB, and SPARC as the most significantly associated with osteosarcoma progression. These data support the involvement of COL1A1, PDGFRB, and SPARC in mtRNA-driven tumorigenesis in osteosarcoma.

COL1A1, encoding the pro-alpha1 chains of type I collagen, which is a fibril-forming collagen abundant in bone, cornea, dermis, and tendon, has also been linked to mitochondrial homeostasis (Dalgleish et al., 2008). In non-cancer models, COL1A1 knockdown increases reactive oxygen species (ROS) and disrupts mitochondrial membrane potential (ΔΨm) (Yuan et al., 2020; Fu et al., 2019). This finding suggests that reduced COL1A1 expression may exacerbate mitochondrial stress initiated by mtRNA depletion, creating a vicious cycle of oxidative damage and energy imbalance in osteosarcoma cells. Building on this concept of mitochondrial dysfunction, PDGFRB emerges as a key mediator of metabolic adaptation. PDGFRB encodes a cell-surface tyrosine kinase receptor that, in osteosarcoma HOS cells, drives aerobic glycolysis through the PI3K/AKT/mTOR/c-Myc pathway while preserving ΔΨm (Tang et al., 2022; An et al., 2024; Xiang et al., 2023). Therefore, when mtRNA is depleted in 143B cells, diminished PDGFRB expression not only impairs this glycolytic shift but also leaves cells unable to compensate for reduced oxidative phosphorylation. Moreover, mitochondrial dysfunction itself may feedback to suppress PDGFRB transcription via reduced mTOR/Akt signaling (Zhang et al., 2007), reinforcing energy stress and inhibiting proliferation under hypoxic conditions.

Extending beyond intracellular metabolism, SPARC serves as a bridge between mitochondrial health and ECM dynamics (Wei et al., 2012; McCurdy et al., 2011). SPARC is a cysteine-rich, acidic matrix-associated glycoprotein that, in C2C12 myoblasts, upregulates mitochondrial proteins UQCRC2 and SDHB via the ILK/AMPK pathway (Melouane et al., 2019; Melouane et al., 2018; Cai et al., 2023) and participates in the mitochondrial unfolded protein response under stress (Gaspar et al., 2023). In mtRNA-deficient 143B tumors, reduced SPARC expression could, therefore, disrupt ECM-mitochondria crosstalk, weaken quality control mechanisms, and facilitate invasive behavior through altered matrix remodeling. These observations support an integrated model: mtRNA loss undermines mitochondrial protein synthesis and function, which in turn suppresses COL1A1, PDGFRB, and SPARC through distinct but interconnected pathways. The downregulation of COL1A1 amplifies ROS and ΔΨm collapse, decreased PDGFRB limits glycolytic compensation, and reduced SPARC disrupts ECM-mitochondria signaling. Collectively, this network drives oxidative stress, metabolic imbalance, and matrix remodeling, promoting osteosarcoma progression under conditions of mitochondrial stress.

## 5 Limitations

This study has limitations. First, pathway enrichment analyses such as GO and KEGG, while informative, may produce false negatives or biased results due to underlying method assumptions and gene correlations (Ge et al., 2022; Tamayo et al., 2016; Glass and Girvan, 2014; Qiu et al., 2021). Consequently, some mitochondrial-related loci adjacent to our identified DEGs may have been overlooked. Second, the sample size of the GSE73120 dataset (143B mtDNA-proficient and rho tumors) was limited, reducing statistical power and generalizability. Finally, although we identified COL1A1, PDGFRB, and SPARC as potential mediators of mtRNA-driven tumorigenesis, experimental validation remains essential. Functional studies, such as loss-and gain-of-function assays, metabolic and ROS measurements, and *in vivo* xenografts, will be crucial to establish causal links and assess therapeutic potential.

## 6 Conclusion

Our analysis of GSE73120 revealed 104 DEGs enriched in extracellular matrix-related pathways, notably connective tissue development and collagen trimer formation. Network analysis and GEPIA validation highlighted COL1A1, PDGFRB, and SPARC as central hub genes linked to mtRNA-dependent tumorigenesis. We propose a model in which mtRNA depletion triggers mitochondrial dysfunction, leading to suppression of these three genes, accumulation of ROS, energetic imbalance, and ECM remodeling, thereby promoting osteosarcoma progression. These insights lay the groundwork for future functional studies and potential therapeutic targeting in mtRNA-related osteosarcoma.

## Data Availability

The original contributions presented in the study are included in the article/Supplementary Material, further inquiries can be directed to the corresponding author.

## Supplementary material

SUPPLEMENTARY TABLE 1 Identification of de-expressed genes in GSE7312.

SUPPLEMENTARY TABLE 2 Functional enrichment analyses of DEGs.

SUPPLEMENTARY TABLE 3 PPI network analysis of DEGs.

## Abbreviations

DEGs, different expression genes; GEO, Gene Expression Omnibus; mtRNA, mitochondrial RNA; NCBI, National Center of Biotechnology Information.
